# Mode jumping of split-ring resonator metamaterials controlled by high-permittivity BST and incident electric fields

**DOI:** 10.1038/srep31274

**Published:** 2016-08-09

**Authors:** Xiaojian Fu, Xinxi Zeng, Tie Jun Cui, Chuwen Lan, Yunsheng Guo, Hao Chi Zhang, Qian Zhang

**Affiliations:** 1 State Key Laboratory of Millimeter Waves, School of Information Science and Engineering, Southeast University, Nanjing 210096, China; 2 Synergetic Innovation Center of Wireless Communication Technology, Southeast University, Nanjing 210096, China; 3 Division of Energy & Environment, Tsinghua University Graduate School at Shenzhen, Guangdong, 518055, China; 4 State Key Laboratory of New Ceramics and Fine Processing, School of Materials Science and Engineering, Tsinghua University, Beijing 100084, China; 5 Center for Terahertz Waves and College of Precision Instrument and Optoelectronics Engineering, Tianjin University, Tianjin 300072, China

## Abstract

We investigate the resonant modes of split-ring resonator (SRR) metamaterials that contain high-permittivity BST block numerically and experimentally. We observe interesting mode-jumping phenomena from the BST-included SRR absorber structure as the excitation wave is incident perpendicularly to the SRR plane. Specifically, when the electric field is parallel to the SRR gap, the BST block in the gap will induce a mode jumping from the LC resonance to plasmonic resonance (horizontal electric-dipole mode), because the displacement current excited by the Mie resonance in the dielectric block acts as a current channel in the gap. When the electric field is perpendicular to the gap side, the plasmonic resonance mode (vertical electric-dipole mode) in SRR changes to two joint modes contributed simultaneously by the back layer, SRR and BST block, as a result of connected back layer and SRR layer by the displacement current in the BST dielectric block. Based on the mode jumping effect as well as temperature and electric-field dependent dielectric constant, the BST-included SRR metamaterials may have great potentials for the applications in electromagnetic switches and widely tunable metamaterial devices.

Metamaterials (MMs) are known as artificially designed periodic structures, which have attracted considerable attentions for two decades^1^,^2^,^3^,^4^,^5^. The electromagnetic (EM) properties of MMs including resonant frequency and EM parameters depend on the structure and the size of the component unit cell^6^,^7^. Based on this principle, some exotic features such as gradient refractive index, negative refraction and high-frequency magnetic resonance are realized, and the potential applications in EM cloak, high frequency magnetism and super lens are also expected^8^,^9^,^10^. Split-ring resonator (SRR) is a kind of typical MM unit structure, which usually has strong resonance and high Q factor. Thus, SRR and its variants have been extensively investigated for application in EM devices such as filters, absorbers and antennas^11^,^12^,^13^,^14^. However, tunable operating frequency is usually needed in consideration of practical application in absorber or filter, which may not be easily achieved with only metallic SRR based MMs.

Besides the metallic MMs, dielectric-material-based abnormal media also focus many research interests. High-permittivity material cubic or rod can generate strong electric dipolar or magnetic dipolar resonances according to the Mie theory^15^,^16^. Ferrites show intrinsic ferromagnetic resonances at microwave frequencies under an applied magnetic field^17^. Compared to metallic MMs, dielectric MMs may have the advantage of tunable working frequency. Some temperature-sensitive materials such as VO_2_, titanates and magnetically tunable ferrites have been introduced to MM systems, resulting in frequency-tunable EM resonances and also tunable negative refraction, absorber, and so on^18^,^19^,^20^,^21^. Nevertheless, the previous mechanisms usually continuously tune the frequency in a limited range without involving the change of resonant mode, which may limit the application in broadband tunable devices and EM switch.

Here, a kind of high-permittivity BST [Ba_0.5_Sr_0.5_TiO_3_] ceramic material is introduced to the gap area of the SRR structure to obtain a BST-included SRR MM. The composite MM is designed at microwave frequencies with an absorber structure including SRR, BST, substrate and metallic back layer, then both simulations and experiments are performed on the MM. Absorption at resonance and tunable resonant frequency can be expected due to the electric-field-tunable dielectric constant of BST. Moreover, some interesting resonant-mode jumping phenomena are observed in this MM, accompanied by remarkable frequency shift. Thus, this new mechanism shines some light to realization of widely tunable absorber and reflective filter as well as reflective EM switch.

## Results and Discussion

**Figure 1:**
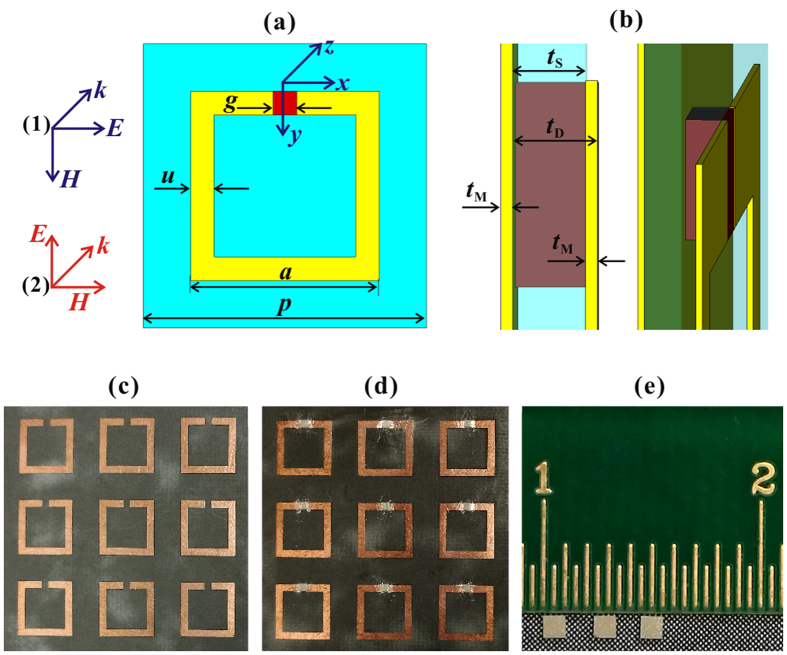
The schematic diagram of the BST dielectric block contained SRR metamaterial and photos of experimental samples. (**a**) the front view of the structure, the incident plane wave have two polarizations, with the electric field parallel (blue axis, ***E***//*x*) or perpendicular (red axis, ***E***⊥*x*) to the ring gap, respectively. (**b**) the magnified left view of the SRR gap and the BST block. The MM structure consists of a copper back layer, a substrate layer of Rogers5880 and the SRR units, whose thickness are *t*_M_ = 0.035 mm, *t*_S_ = 0.254 mm, and *t*_M_ = 0.035 mm, respectively. In the SRR gap region, a through-hole is structured on the substrate, and then the BST block with a thickness of *t*_D_ = *t*_S_ + *t*_M_ = 0.289 mm is embedded into the hole, in touch with both the metallic back layer and the SRR layer. (**c**),(**d**) the fractional photos of 10 × 10 SRR and SRR-D arrays. (**e**) the photo of BST dielectric block with a dimention of 1.0 × 1.0 × 0.289 mm^3^.

Figure 1 shows the schematic diagram of the BST dielectric material included SRR absorber unit (denoted as SRR-D) and photos of experimental samples. As shown in Figs 1(a~d), SRR and SRR-D arrays are designed and fabricated on a Rogers-5880 dielectric substrate. The thickness of Rogers-5880 is selected as 0.254 mm and the dielectric constant is 2.20 with a loss factor of tan*δ* = 0.0009. The back layer and functional layer are copper film with a thickness of 0.035 mm. Some optimized geometric parameters of the metallic structure are as follows: the periods along both directions are *p* = 12 mm, the size of square SRR unit is *a* = 8 mm, the line width of the copper ring is *u* = 1 mm, while the width of gap is *g* = 1 mm. A through-hole is processed on the dielectric substrate at the SRR gap, and a BST dielectric block (red block in Fig. 1(a), whose photo is shown in Fig. 1(e)) with the same size of 1.0 × 1.0 × 0.289 mm^3^ is embedded into the hole, contacting with both the back layer and the SRR gap, as shown in Figs 1(b,d). The dielectric constant of BST ceramic in the investigated frequency region is around *ε* = 1600 + i 4.8^15^. Besides, as can be found in Fig. 1(a), the excitation plane is incident along *z* axis (***k***//*z*), however, two different polarizations are considered, with the electric field component parallel ((1) ***E***//*x*) and perpendicular ((2) ***E***⊥*x*) to the ring gap respectively.

**Figure 2:**
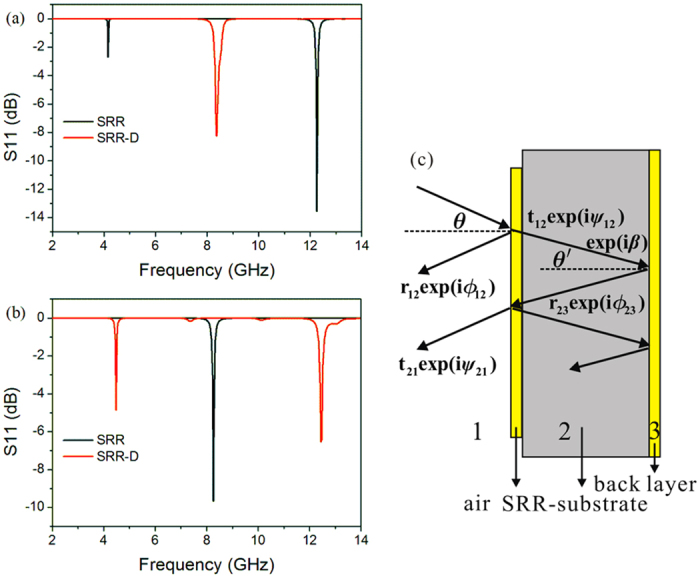
The simulation results of reflection parameter S11 and schematic diagram of destructive interference in SRR absorber structure, in which the electric field component is parallel (**a**) and perpendicular (**b**) to the gap, respectively.

Figure 2 presents the simulation results of reflection parameters S11 for both SRR and SRR-D. Because the low order resonant modes locate between 2 GHz and 14 GHz, we just show the reflection spectra in this frequency region. For SRR, there are three reflection dips observed. As the electric field is parallel to gap (Fig. 2(a)), there are two obvious resonant dips locating at 4.16 GHz and 12.26 GHz, of which the latter one has strong absorption up to −13 dB. However, the reflection curve of SRR-D is totally different since only one dip is observed at 8.38 GHz and the absorption is also weakened. If we rotate the polarization of incident plane wave for 90° to make the electric field perpendicular to gap, the reflection parameters change a lot as shown in Fig. 2(b). For SRR, the resonant frequency is 8.26 GHz, while there are two remarkable resonant dips for SRR-D, with respective frequencies of 4.48 GHz and 12.44 GHz.

For the SRR absorber structure (transmission is zero), the reflection dip can be explained with the destructive interference mechanism^14^,^22^. If we successfully denote air, SRR-substrate and back layer with 1, 2 and 3 (Fig. 2(c)), the total reflection is the superposition of the reflection from 1–2 and 2–3 interfaces. And hence, the phase difference can be expressed as


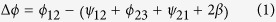


For the case of normal incidence, *β* = *nk*_0_*d*, where *n* and *d* represent the refractive index and thickness of the substrate respectively, and *k*_0_ is the wave number in free space. As Δ*ϕ* = (2 *m* + 1)π (*m* is an integer) and the two reflection waves with close amplitude, the antireflection condition will be satisfied. In the SRR absorber system, *ϕ*_12_ will have an abrupt change when SRR is at resonance, and if the total phase difference satisfies the antireflection condition (which can be realized through tuning *n* and *d*), there will be a reflection dip at the resonant frequency, while the energy is confined in the substrate by SRR and back layer, and consumed by the resonant absorption and the lossy materials.

**Figure 3:**
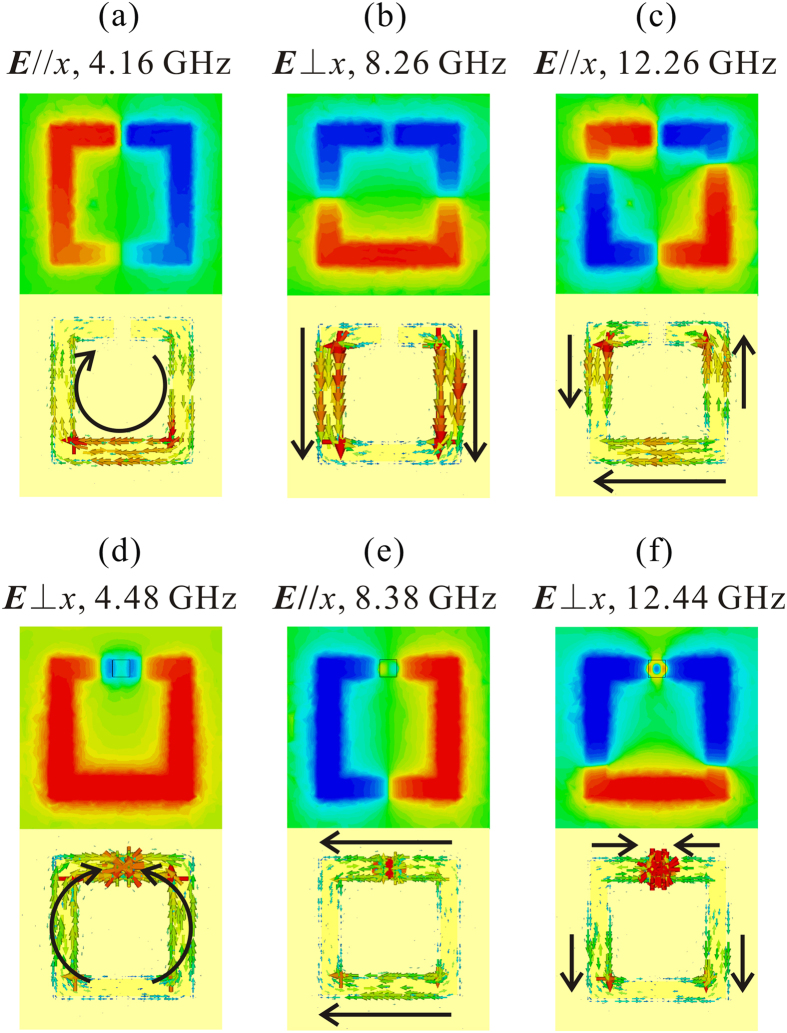
The electric field (*E*_z_ component) distribution in the central *xy* plane of the substrate and surface current distribution of SRR (**a–c**) and SRR-D (**d–f**) at the resonant frequencies.

Next, the resonant modes can be assigned according to the electric field and surface current distributions in Fig. 3. A *xy* plane at the center of the dielectric substrate (0.127 mm lower than the SRR layer) is selected to investigate the near field. As can be seen in Fig. 3(a~c), the resonance modes at 4.16 GHz and 12.26 GHz show the characteristics of horizontal electric dipole and electric quadrupole modes respectively, while the resonance at 8.26 GHz can be attributed to a vertical electric dipole^23^,^24^. From the surface current distribution, we may further find that the mode at 4.16 GHz is a typical *LC* resonance, because of the circular current flow in the ring. Moreover, for the second mode at 8.26 GHz, the currents concentrate on the surface of the two vertical SRR arms, which are parallel and change in phase, and thus it is a plasmonic resonant mode, and can be equivalent to the resonance of two metallic lines with the same geometry. The third mode is a horizontal electric quadrupole mode, while for the case of ***E***⊥*x*, the very weak high order modes are above 14 GHz and are not shown in the figure.

When a high-permittivity ceramic block is embedded into the gap and keeps electrically contact with both the copper back layer and SRR layer, the resonant modes of the composite MM change a lot. According to the electric field distribution in Fig. 3(e), the resonance at 8.38 GHz can be ascribed to a horizontal electric dipole, similar with the corresponding mode of SRR at 4.16 GHz (Fig. 3(a)), except of a frequency blue shift. However, the current distribution provides totally different and very interesting information: the current flow in the SRR is not a circular type, but concentrates on the surfaces of the top and bottom arms with the same flow direction. And therefore, the mode should be ascribed to a plasmonic resonance instead of the *LC* resonance mode. It is worth noting that there are currents flowing through the BST dielectric block, and hence the block acts as a current channel, which results in the formation of a closed metallic ring and the absence of *LC* resonance mode. As such, the ring can be equivalent to two horizontal metallic lines and exhibits close resonant frequency to the vertical electric dipole mode of SRR at 8.26 GHz. Additionally, the horizontal electric quadrupole resonance does not appear in the investigated frequency region for SRR-D. It may lie above 14 GHz and have a weak strength according to the experience of vertical quadrupole mode in SRR. The dips at 4.48 GHz and 12.44 GHz are induced by the vertical electric dipole and electric quadrupole, respectively, both of which are contributed jointly by the BST block and the SRR. For the lower frequency dipolar mode, the surface currents start at the bottom of SRR, flow along the arm of SRR, and end at the BST block (or vice versa), and two branches are symmetric about *y* axis. For the higher frequency one, the current flows will be more complicated. There are two current directions, one of which is from the middle part to the bottom part in SRR, while the other is from the middle SRR to the dielectric block, and the currents near the BST block point from the outer to the inner (vice versa). It is also shown that the BST block works as a current concentrator for both modes because the current density on the surface of block is much larger than that of SRR. Note that the resonant modes of SRR-D with ***E***⊥*x* cannot be explained with only closed ring assumption and we will give detailed illustrations later.

**Figure 4:**
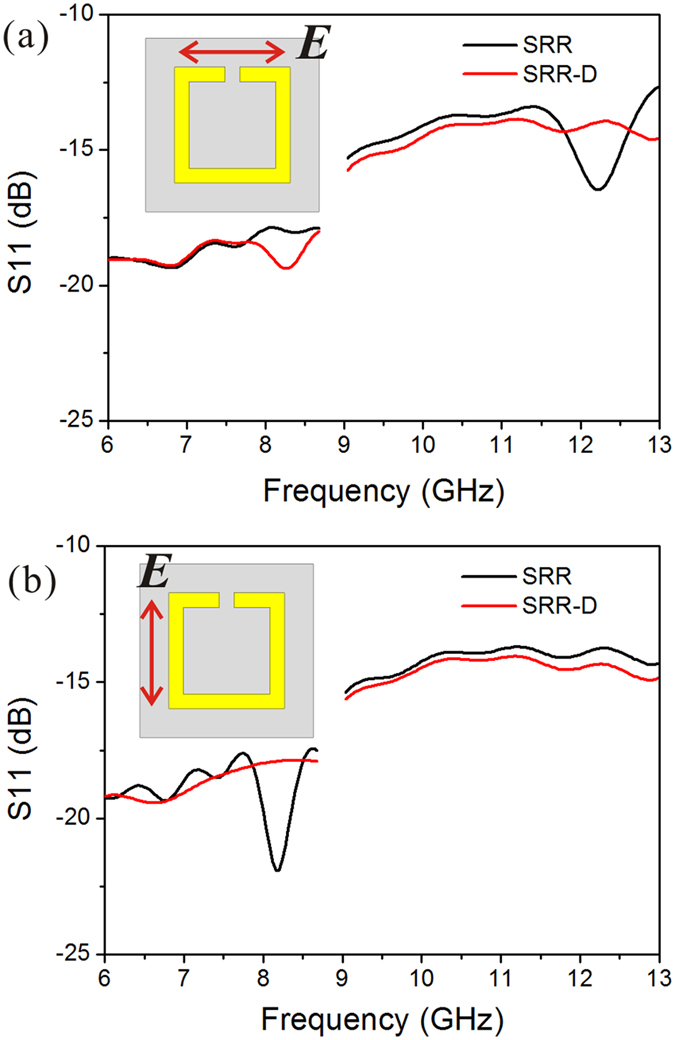
The experimental results of reflection parameter S11 for SRR and BST dielectric block contained SRR, in which electric field component is parallel (**a**) and perpendicular (**b**) to the gap, respectively.

Figure 4 presents the experimental results of S11 between 6 and 13 GHz. Because the size of the sample is 12 × 12 mm^2^, the low frequency portion is not measured in this work (the needed antenna aperture is much larger than the sample and the reflection signal will be very weak). As shown in Fig. 4(a), the electric field is parallel to the SRR gap (***E***//*x*). A reflection dip is observed at 12.20 GHz (center frequency) for SRR, very close to the simulation value of 12.26 GHz. As for SRR-D, a resonant dip at 8.28 GHz is observed, which is also in consistent with the simulation result of 8.38 GHz. When the electric field component gets perpendicular to the gap (***E***⊥*x*, see Fig. 4(b)), a vertical electric dipole mode appears at 8.16 GHz, in agreement with the simulated value of 8.26 GHz. However, the expected reflection dip around 12.44 GHz for SRR-D is absent. The possible reason is that the amplitude is too small. Actually, the measured amplitudes of reflection dips for both SRR and SRR-D are much smaller than the numerical results. It can be partially explained by the band broadening due to the size variation and dielectric loss, especially for the BST block, the loss factor is not lower than 0.03. The small area of the sample compared to the antenna may also lead to the low measurement accuracy of S parameters.

To further understand the physical mechanism of mode jumping in the SRR-D sample, we will change the thickness of the BST block and its distance to the metallic back layer.

**Figure 5:**
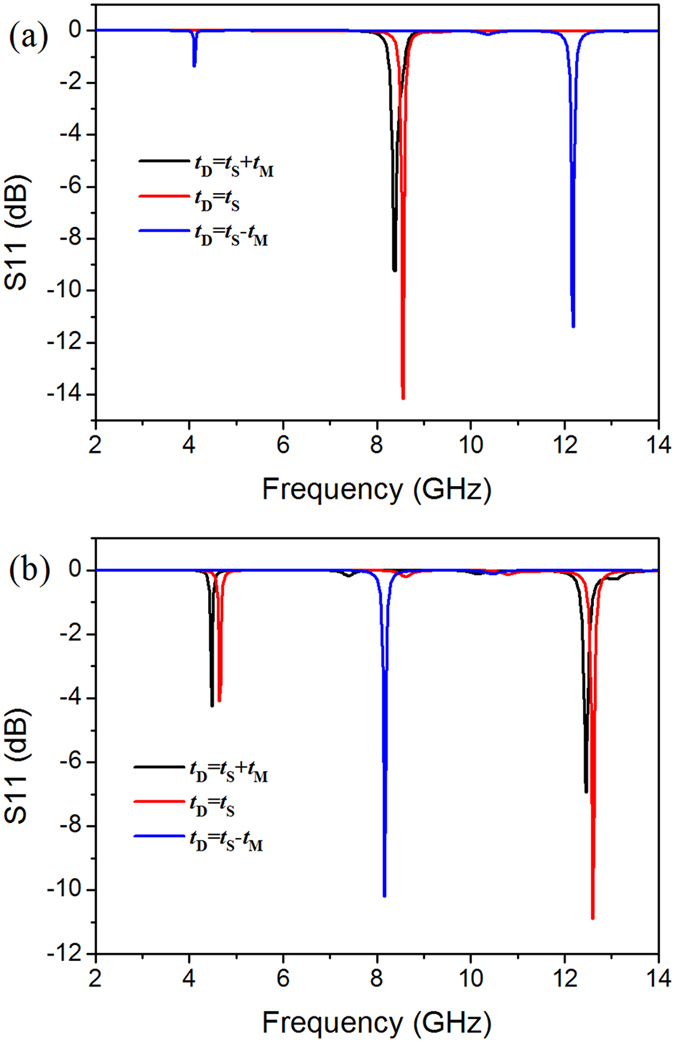
The simulation results of reflection parameter S11 for SRR-D with different block thickness, in which electric field component parallel (**a**) and perpendicular (**b**) to the gap, respectively.

First, we keep the BST block contact with the copper back layer, and then reduce its thickness *t*_D_ from *t*_S_ + *t*_M_ to *t*_S_ and *t*_S_ − *t*_M_ (the BST block separates with the SRR layer), in which *t*_S_ and *t*_M_ denote the thickness of substrate and SRR layer respectively. It is found from Fig. 5 that the resonant modes of SRR-D are similar with SRR (mode jumping does not occur for both electric field polarizations) as if the BST block does not contract with the SRR layer. Specifically, when *t*_D_ = *t*_S_ − *t*_M_, the structure shows two resonant dips at 4.10 GHz and 12.18 GHz at the direction of ***E***//*x*, and a resonant mode at 8.16 GHz at ***E***⊥*x*. However, once the BST block contacts with SRR as *t*_D_ = *t*_S_, the mode jumping appears: the horizontal mode shifts to around 8.55 GHz, while two dips appear at about 4.65 GHz and 12.60 GHz as ***E***⊥*x*, similar to the case of SRR-D with *t*_D_ = *t*_S_ + *t*_M_.

**Figure 6:**
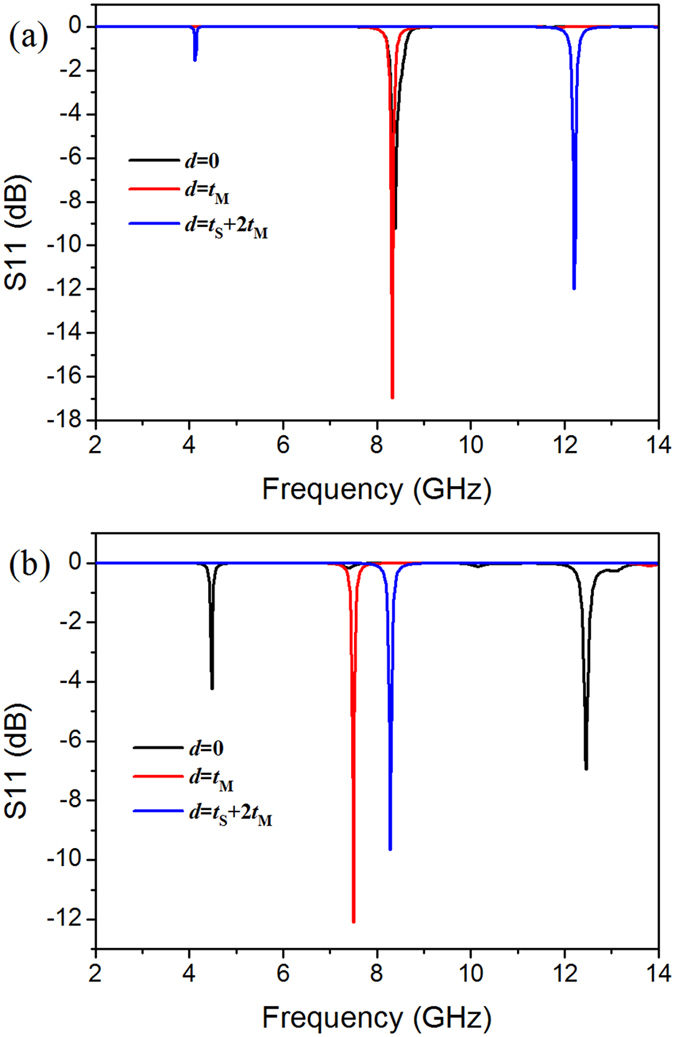
The simulation results of reflection parameter S11 for SRR-D with different back layer-block distance, in which electric field component parallel (**a**) and perpendicular (**b**) to the gap, respectively.

Then, we keep the thickness of the block a constant *t*_D_ = *t*_S_ + *t*_M_, and try to vary the distance of block with the copper back layer *d* from 0 to *t*_M_ (the BST block separates with the back layer, but still contact with the SRR layer) and *t*_S_ + 2 × *t*_M_ (the BST block separates with the SRR layer). The results are presented in Fig. 6. As can be seen, when the BST block sperates with the back layer, the horizontal mode keeps at about 8.32 GHz, similar with SRR-D, whereas the vertical mode jumps to 7.50 GHz. Then, the block seperates with SRR layer as *d* = *t*_S_ + 2 × *t*_M_, the horizontal mode also returns to the case of SRR, with two dips at 4.12 GHz and 12.20 GHz.

Thus, we may draw a conclusion that the mode jumping conditions for the horizontal and vertical modes are different: the electrically contact of BST dielectric block with SRR layer induces a mode jumping at ***E***//gap (***E***//*x*), that is because the current channel effect of the BST block as previously discussed. However, the mode jumping at ***E***⊥*x* happens only if the block contacts with the back layer and SRR layer simultaneously, which indicates the BST block acts as a current channel between the two metallic layers. That is, the high-permittivity BST dielectric block can be equivalent to a conductor for both electric field polarizations. In order to verify this assumption, we do a simulation with copper block instead of the dielectirc block.

**Figure 7:**
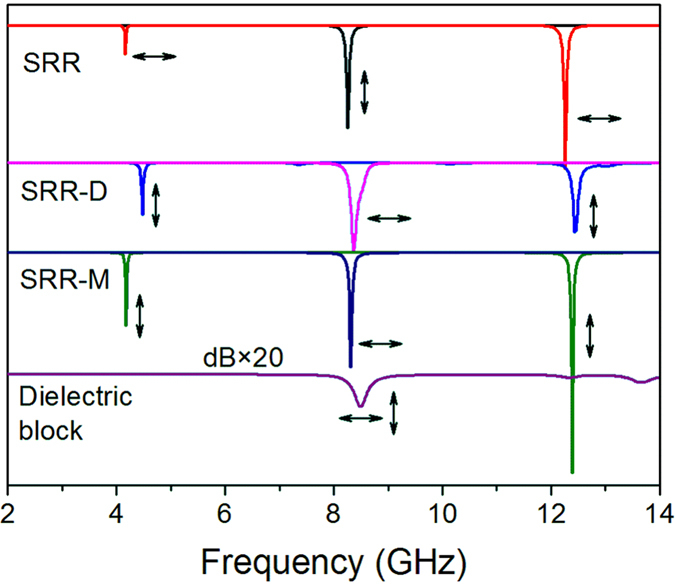
The resonant modes of SRR, SRR-D, SRR-M and BST dielectric block.

**Figure 8:**
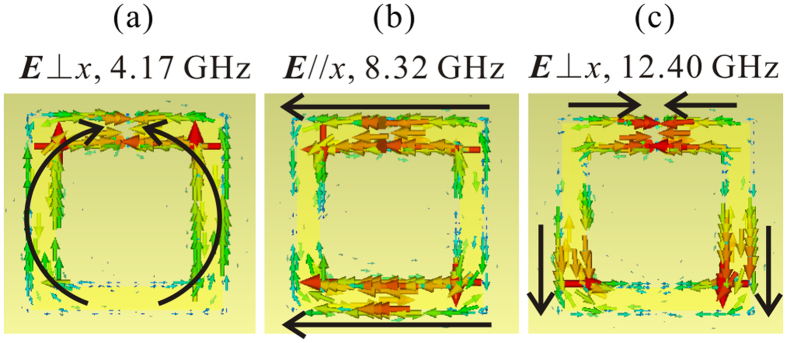
The surface current distribution of metallic block included SRR (SRR-M) at the resonant frequencies.

Figure 7 shows the resonant modes of SRR, SRR-D and SRR-M (the BST dielectric block is instituted by a metallic copper block with the same size). Comparing the reflection dips of SRR-D and SRR-M, we may find their resonant modes are the same for both two electric field polarizations, although the resonant frequencies shift to some extent. Figure 8 shows the surface current distribution of SRR-M, which is the similar with the current distributions of SRR-D in Fig. 3. However, the copper block does not exhibit current concentrating effect as the BST block does. Thus, the BST block acts as a current channel. Specifically, when the electric field is parallel to the gap, the block bridges the gap and a closed ring forms; as the electric field perpendicular to the gap, the block connects the back layer and SRR layer, and the mode jumping is contributed by the back layer, SRR and dielectric together.

Actually, as we know, dielectric materials are usually electrically insulated. How can they behave as current channel? We think the displacement current induced by Mie resonance mechanism may account for it^15^. The scattering field coefficients of a dielectric sphere can be expressed by the following equations^25^.


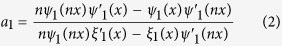



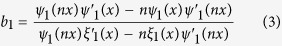


where *a*_1_ and *b*_1_ represent the scattering coefficients of the electric dipole and the magnetic dipole respectively, *n* is the refractive index of dielectric sphere, while *ψ*_1_ and *ξ*_1_ are the Riccati-Bessel functions.

We remove the SRR layer in SRR-D and do a simulation with separate BST block, whose reflection is also presented in Fig. 7 for comparison. Because the block is isotropic in *xy* plane, the S parameters are equal for both two polarizations. As can be seen, most of the reflection dips are very small (<0.2 dB) and a relatively strong resonance is observed at 8.50 GHz. It suggests that the strong surface currents in BST block observed in Fig. 3(d~f) are related to the electromagnetic coupling between SRR and dielectric block. For ***E***//*x*, the weak Mie resonance at 8.50 GHz (it is attributed to an electric dipolar resonance according to the field distribution) induces a displacement current flow along *x* axis in the SRR gap which bridges the gap and leads to the mode jumping from *LC* resonance to plasmonic resonance; then, the plasmonic resonance in the closed ring at 8.38 GHz enhances the localized electric field in the gap and results a stronger resonance in the BST dielectric block and also a larger displacement current, which may in turn effect on the plasmonic resonance in the metallic ring. For ***E***⊥*x*, the physics will be a little different. The block also bridges the back layer and SRR layer with displacement current (which means the block is in an excited resonant state), however, the resonant mode is contributed by all of them. For the mode at 4.48 GHz, the electric dipole is constituted by SRR and dielectric, while the electric quadrupole resonance at 12.44 GHz is also the joint contribution of them, since separate BST block will not response to these frequencies.

## Conclusions

In summary, the mode jumping effect in high-permittivity BST included SRR absorber structure was studied in this work. It is demonstrated by both simulations and experiments that the BST dielectric block can be equivalent to a metallic block and acts as a current channel due to the displacement current induced by the Mie resonance. As the electric field is applied parallel to the SRR gap, the block bridges the gap by displacement current and forms a closed ring, resulting the mode jumps from *LC* resonance to plasmonic resonance. For the perpendicular electric field, the induced current in the dielectric block connects the metallic back layer and SRR, and then generates two new modes instead of the original plasmonic resonance mode. As the absorption frequency exhibits remarkable shift for different contacting conditions of SRR and BST, a kind of reflective EM switch can be designed.

Note that the dielectric constant of BST ceramic is sensitive to temperature and applied electric field. According to previous studies, the tuning range of dielectric constant reaches higher than 50% under applied field^26^, which indicates a widely tunable resonant frequency of the composite SRR metamaterials. Thus, the dielectric material included SRR may also have potential application in tunable EM devices such as absorber and reflective filter. Moreover, the BST ceramics or films remain a high permittivity at terahertz frequencies (up to 300 at 0.5 THz)^27^, and hence the interesting results in this work can also extend to the terahertz regime.

## Methods

The numerical simulation of the SRR resonant structure was processed using the CST Microwave Studio. Broadband reflection parameters between 0 and 20 GHz were calculated and the resonant frequencies have been extracted from the reflection spectra. It is noted that the transmission is zero because of a thick enough metallic back layer. In order to clarify the resonant modes, the near-field distribution and surface current distribution were also obtained. The plane-wave reflection parameters of SRR were measured with vector network analyzer (Agilent N5230C) and standard gain horn antennas (15 dB) of 5.38~8.17 GHz and 8.20~12.4 GHz. The plane wave is incident perpendicular to the *xy* plane, whereas the orientations of electric field component are set to be parallel to the SRR gap (***E***//*x*) or perpendicular to it (***E***//*y* or ***E***⊥*x*), respectively.

## Additional Information

**How to cite this article**: Fu, X. *et al*. Mode jumping of split-ring resonator metamaterials controlled by high-permittivity BST and incident electric fields. *Sci. Rep.* **6**, 31274; doi: 10.1038/srep31274 (2016).
